# Sorafenib Induced Hand Foot Skin Rash in FLT3 ITD Mutated Acute Myeloid Leukemia-*A Case Report and Review of Literature*

**DOI:** 10.4084/MJHID.2014.016

**Published:** 2014-02-17

**Authors:** Jayastu Senapati, Anup J. Devasia, Abhijeet Ganapule, Leni George, Auro Viswabandya

**Affiliations:** 1 Department of Clinical Haematology, Christian Medical College and Hospital, Vellore-632004. India.; 2 Department of Dermatology, Christian Medical College and Hospital, \Vellore-632004. India.

## Abstract

Sorafenib is a novel small molecule multiple kinase inhibitor which has been used for metastatic renal cancer, hepatocellular cancer. Sorafenib induced skin rash has been discussed as a side effect in trials in both, FLT3 wild type and mutated acute myeloid leukemia (AML), as monotherapy or as combination with other chemotherapeutic agents. We describe a patient with FLT 3 ITD mutated AML, who was started on adjunctive Sorafenib therapy. Skin reactions manifested as NCI Grade III palmoplantar erythrodysesthesia (PPE), requiring drug discontinuation. Several pathogenic mechanisms have been implicated in Sorafenib induced skin reactions, but none has been conclusively proven. While treatment options are varied for early stage skin reactions, drug discontinuation remains the only possible therapy presently for severe grade skin reaction.

## Introduction

Sorafenib induced skin rash has been widely described in context to its use in advanced renal and hepatocellular cancer.^1^–^4^ Here we describe a case of a classical Sorafenib induced hand foot skin rash (HFSR) in a patient with acute myeloid leukemia (AML).

## Case Report

**Figure 1:**
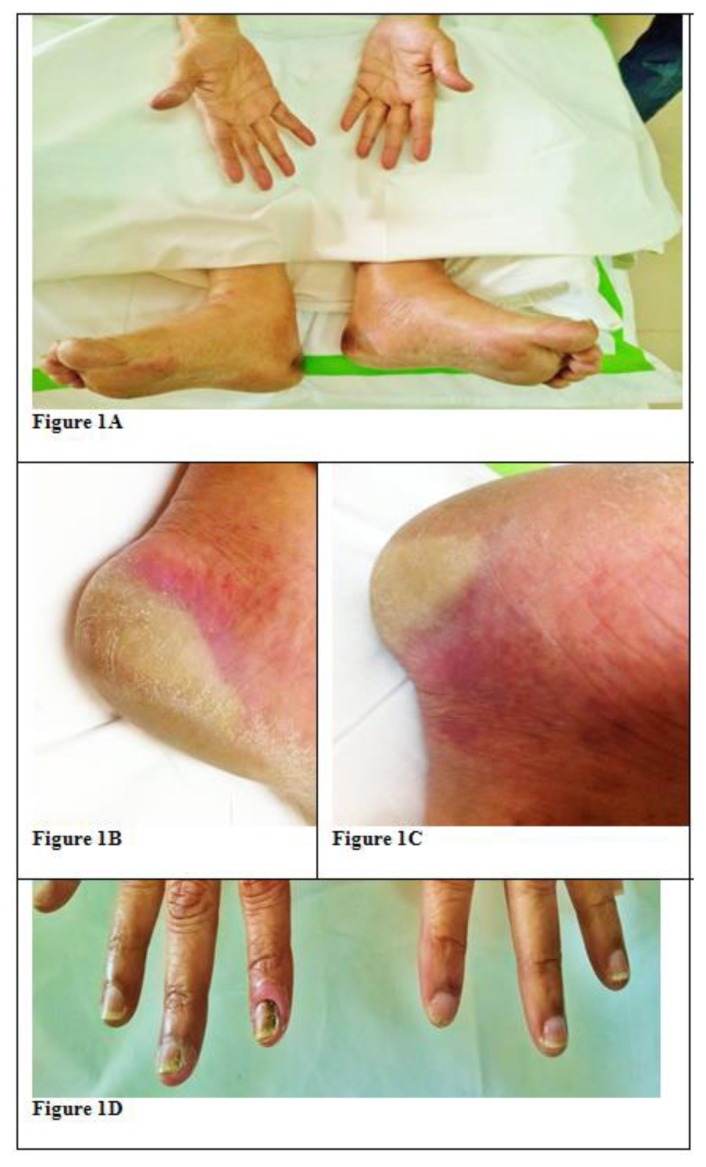


A 63 years old lady, with no significant history was diagnosed with AML with myelodysplasia related changes. Her cytogenetic analysis revealed normal karyotype and molecular analysis showed positivity for FLT3 ITD and NPM 1 frame shift mutation. She was initially started on Azacytidine based chemotherapy given her age and poor general condition. The family had been made aware of the poor outcome and had opted for best supportive therapy with Azacytidine. After 2 cycles of the same as there was disease progression, she was started on cytosine and daunorubicin chemotherapy (5+2) with Sorafenib as an adjunct therapy at a dose of 400 mg twice daily. Ten days after starting Sorafenib she complained of bilateral heel pain while walking, associated with paresthesia and erythema of the skin which increased over the next 2 days with formation of bulla and hyperesthesia. Similar rashes were also noticed over her palms with discoloration of nail beds (Figure 1).

**Figure 2:**
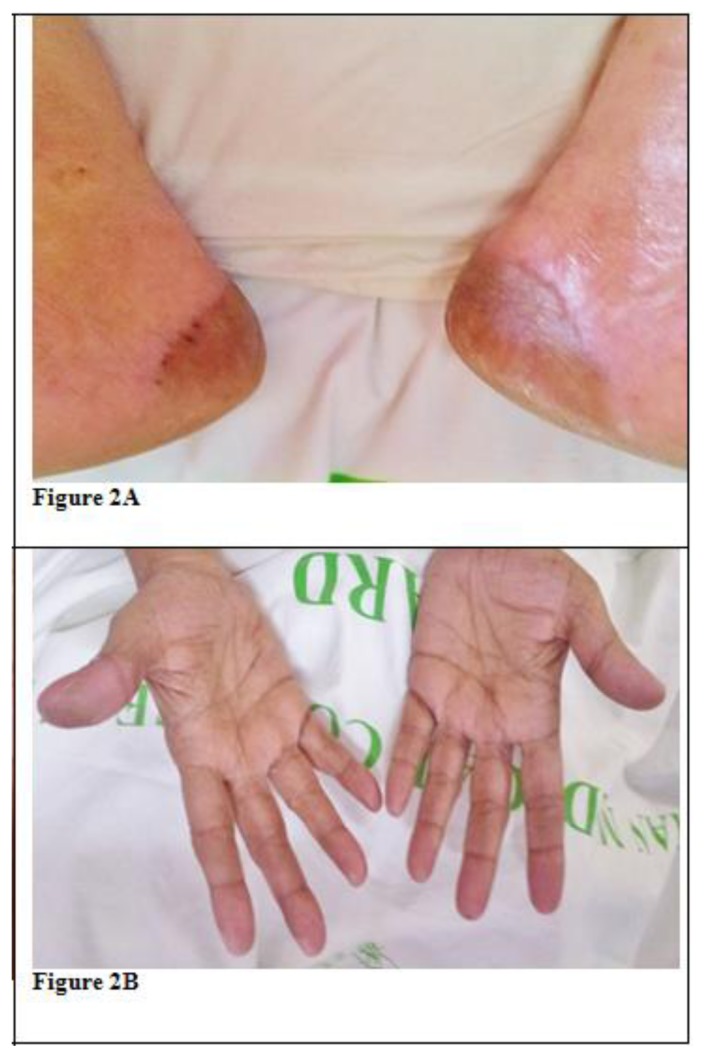


She had difficulty in performing her activities of daily life. A dermatology opinion was sought, and a diagnosis of Sorafenib induced HFSR was made. She was staged as NCI Grade III/WHO Grade IV and was started on topical tacrolimus and clobetasol along with analgesics. In view of progressive skin manifestations and inadequate pain relief with analgesics, Sorafenib was discontinued on day 12 of therapy. Three days after stopping Sorafenib there was decrease in heel pain, and the erythema decreased with healing of blisters. She became NCI grade II within 3 days of stopping Sorafenib and Grade I within 6 days (Figure 2). She became asymptomatic by another 7 days and was ambulant normally.

## Discussion

Sorafenib is a novel multiple tyrosine kinase inhibitor which has shown efficacy in FLT3 ITD mutated AML, alone or in addition to other cytotoxicity drugs.^5^–^11^ We discuss here the important clinical features of HFSR and management algorithm. HFSR have been described as the most common side effect with the use of Sorafenib in solid tumours. A systematic analysis in solid tumours showed an overall incidence of all grade Sorafenib HFSR of 33.8 % with 8.9% being Grade III.^12^ HFSR has been described as one of the commonest toxicities of Sorafenib; in several trials using Sorafenib as a single agent or as add-on therapy to other cytotoxic therapies in leukemia. Table 2 lists the major trials and the frequencies of HFSR in study patients. This to our knowledge is the first reported case, of Sorafenib induced HFSR, outside trial data, in a case of acute myeloid leukemia where Sorafenib was used as add on therapy along with standard chemotherapy.

HFSR associated with Sorafenib belongs to the spectrum of PPE, which is associated with the use of several cytotoxic chemotherapy drugs, the most common being capecitabine and 5-fluoro-uracil. Table 3 lists the common cytotoxic medications associated with PPE. However, the HFSR associated with Sorafenib therapy focally affects the weight and friction bearing acral surfaces, unlike the classic hand foot syndrome (HFS) reported with other chemotherapeutic agents.^13^ HFS usually presents as diffuse erythema of palms and soles that does not have predilection to areas of friction or trauma.^14^

Sorafenib induced HFSR can present in varied forms, ranging from mild acral erythema to severe hyperesthesia with desquamation of the skin leading to severe morbidity and drug discontinuation. This skin rash has classically been graded by the National Cancer Institute (NCI) and World Health Organisation (WHO) grading systems (Table 1). Several mechanisms have been implicated in the pathogenesis of Sorafenib induced skin rash. Direct cytotoxicity by increased concentration of the drug in the palmo-plantar eccrine glands has been postulated.^15^ Inhibition of vascular endothelial growth factor (VEGF) and platelet derived growth factor receptors (PDGFR) seem to play a role.^16^ Due to inhibition of growth and repair pathways mediated by the above mentioned pro-angiogenic receptors, areas subjected to high pressure and friction are prone for HFSR with Sorafenib.^17^ VEGF seems to play an important role as combination of Bevacizumab which is a specific antibody against VEGF, with Sorafenib increases the incidence of HFSR, while HFSR has not been described in Bevacizumab monotherapy.^13^

Therapy depends on the stage of HFSR. While early stage lesions require only regular dermatological evaluation, emollients, adequate protection from environment; advanced stage lesions require topical immunomodulators, dose modification and often discontinuation of the drug (Table 4).^16^

In our patient, the progression of cutaneous symptoms from NCI Grade I to NCI grade III over a span of 2 days, even with adequate local immunomodulatory therapy and analgesics, required discontinuation of the drug. There was a significant response to discontinuation of the drug, and her symptoms downgraded to Grade I by 4 days. It is difficult to say whether the daunorubicin and cytosine had any role in her cutaneous symptoms, but looking at the distribution pattern of the skin rash and the temporal profile of the Sorafenib administration and discontinuation in conjunction with her cutaneous findings, it is fairly clear that it is primarily due to Sorafenib.

## Conclusions

Sorafenib induces HFSR less frequently in acute myeloid leukemia than in solid cancers treated together with Bevacizumab. HFSR results in significant morbidity, and dose modification including drug discontinuation remain the only option for high grade HFSR.

## Figures and Tables

**Table 1:** 

Grade	1NCI grading	WHO grading
I	Minimal skin changes or dermatitis (erythema, edema or hyperkeratosis) without pain.	Dysesthesia, paresthesia, tingling of hand and feet
II	Skin changes (Peeling, blisters, bleeding, edema or hyperkeratosis) with pain; limiting instrumental ADL.	Discomfort on holding objects and walking, painless swelling and erythema.
III	Severe skin changes (Peeling, blisters, bleeding, edema or hyperkeratosis) with pain; limiting self care ADL	Painful erythema of palms and soles with periungual edema and swelling
IV	N/A	Desquamation, ulceration or blistering with severe pain.

1 Adapted from NCI CTCAE v 4.0 for Hand foot skin syndrome

**Table 2:** 

Author	Study type	FLT3 status	Dose	Other cytotoxic medications	Incidence of HFSR		
**Ravandi et al7** **N=51**	Phase I/II	Both	400 mg BDx7 days	Idarubicin. Cytosine	Grade 1/2	4	
					Grade 3	2	

**Inaba et al**^20^ **N=12** **AML=11** **ETP ALL=1**	Phase I	Positive	200mg/m^2^	Clofarabine, Cytosine	Grade1/2	5	
					Grade 3	3	
					#Stratum 1 (N=10)	2	
					#Stratum 2 (N=2)	1	

**Borthakur et al5** **N=51** **AML=48** **CMML=2** **Biphenotypic leukaemia=1**	Phase I	Both	Schedules: A:5 days every week for 21 days B:Days 1–14 every 21 days	Nil	Grade 1/2	A	B
					200 mgBD	0/3	1/3
					600 mgOD	0/5	1/3
					400 mgBD	0/15	1/7
					600mg BD	1/8	1/6
					Grade 3	A	B
					200 mgBD	0/3	0/3
					600 mgOD	0/5	0/3
					400 mgBD	0/15	0/7
					600mg BD	0/8	0/6

**Ravandi et al6**	Phase II	Positive	400 mg BD	Azacytidine	Grade 1/2	7%	
					Grade 3	0%	

**Serve et al**^21^ **N=197** **(Sorafenib arm=102)**	RCT	Both	400mg BD	Cytosine/Daunorubicin based high dose chemotherapy	Grade ½	Not known	
					Grade 3	3	

**Metzelder et al11** **N=65**	Retrospective analysis	Both	**Allo SCT group**-600 mg daily median dose **Chemotherapy group**-486.5 mg daily median dose	Nil	Grade 1/2	5	
					Grade 3	3	

**Metzelder et al10** **N=6 (3 before Allo SCT, 3 relapsed after Allo SCT)**	Non randomized clinical trial	Positive	Maximum 800 mg daily	Nil	Grade1/2	1	
					Grade 3	0	

**#Stratum 1-**Sorafenib administered alone Days 1–7 and days 8–12 concurrently with Clofarabine 40 mg/m^2^ and Cytosine 1gm/m^2^. Single agent Sorafenib continued thereafter till Day 28 if tolerated.

**Stratum 2-**Clofarabine used at a lower dose of 20 mg/m^2 (^Patients who underwent transplantation within prior 6 months, or history of fungal infection in prior 1 month).

AML-Acute myeloid leukemia; ETP ALL-Early T cell precursor acute lymphoblastic leukemia; CMML-Chronic myelomonocytic leukemia; Allo SCT-Allogenic stem cell transplantation; OD-once daily; BD-twice daily; RCT-Randomized controlled trial.

**Table 3:** Common cytotoxic drugs associated with Palmoplantar erythrodysesthesia

Cytotoxic drugs^18^,^19^	Targeted anticancer drugs^2^	

5-Fluoro-uracil	Sorafenib	***Classical HFSR***
		
Capecitabine	Sunitinib	

Vinorelbin	Cetuximab	

Doxorubicin (Liposomal more commonly associated than plain formulation)	Panitumumab	

Irinotecan	Erlotinib	

Cytosine	Lapatinib	

Docetaxel		

**Table 4:** 

HFSR Severity	Intervention	Sorafenib dose modification
***No HFSR***	Maintain frequent contact with physician to ensure early diagnosis of HFSR	
Therapy initiation	Full-body skin examination, pedicure, evaluation by orthotist; wear thick cotton gloves and/or socks; avoid hot water, constrictive footwear, and excessive friction If symptoms develop at 2-week clinical evaluation or within first month, proceed to next step	
***GRADE 1***	Maintain current dose of Sorafenib; monitor patient for change in severity	No Sorafenib dose modification required
Numbness Tingling Dysesthesia Paresthesia Painless swelling Erythema Discomfort of hands or feet No interference with ADL	Avoid hot water; use moisturizing creams for relief; wear thick cotton gloves and/or socks; use a 20%–40% urea-based cream to aid exfoliation If symptoms worsen after clinical evaluation at 2 weeks, proceed to next step	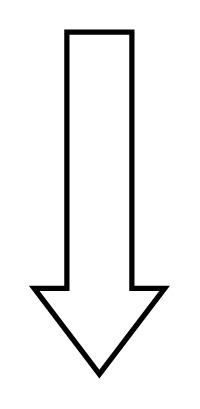
***GRADE 2***	Dose reduction to 50% of dose for 7–28 days	**First occurrence** Promptly institute supportive measures such as topical therapy for symptomatic relief and consider a dose decrease for a minimum of 7 days and up to 28 days: Sorafenib, 400 mg OD (4 weeks on–2 weeks off cycles) If toxicity resolves to grade 0 or 1 after dose reduction, increase to registration dose: Sorafenib, 400 mg BD (4 weeks on–2 weeks off cycles). If toxicity does not resolve to grade 0 or 1 despite dose reduction, interrupt Sorafenib treatment for a minimum of 7 days and until toxicity has resolved to grade 0 or 1. When resuming treatment after dose interruption, begin at reduced dose: Sorafenib, 400 mg daily (4 weeks on–2 weeks off cycles). If toxicity is maintained at grade 0 or 1 at reduced dose for a minimum of 7 days, increase to registration dose
Painful erythema Swelling of hands and/or feet Interferes with patient’s ADL	Treat as with grade 1 toxicity, with the following additions: clobetasol 0.05% ointment, 2% lidocaine, codeine, pregabalin for pain; follow dose modifications as mentioned. If symptoms worsen after clinical evaluation at 2 weeks, proceed to next step	**Second or third occurrence** Same as for first occurrence; upon resuming Sorafenib treatment, decrease dose by one dose level (Sorafenib, 400 mg OD or 400 mg QOD); decision whether to re-escalate dose should be based on clinical judgment and patient preference **Fourth occurrence** Decision whether to discontinue Sorafenib treatment should be based on clinical judgment and patient preference 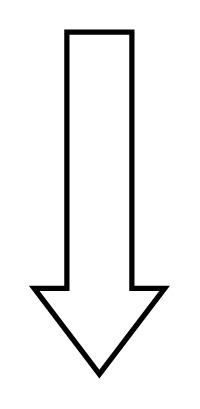
***GRADE 3***	Interrupt treatment for 7 days and until improvement to grade 0–1	**First occurrence** Institute supportive measures such as topical therapy for symptomatic relief and interrupt Sorafenib treatment for a minimum of 7 days and until toxicity has resolved to grade 0 or 1. When resuming treatment after dose interruption, decrease by one dose level (Sorafenib, 400 mg OD or 400 mg QOD.) If toxicity is maintained at grade 0 or 1 at reduced dose for a minimum of 7 days, increase by one dose level (Sorafenib, 400 mg BD or 400 mg OD)
Moist desquamation Ulceration Blistering Severe pain of hands and/or feet Patient unable to perform Activities of daily living	Treat as with grades 1 and 2 Follow dose modifications listed in Table	**Second occurrence** Same as for first occurrence; upon resuming treatment, decrease dose by one dose level (Sorafenib, 400 mg OD or 400 mg QOD); decision whether to re-escalate dose should be based on clinical judgment and patient preference **Third occurrence** Decision whether to discontinue Sorafenib therapy should be based on clinical judgment and patient preference

*ADL-Activities of daily living; HFSR-Hand-foot skin reaction; BD-twice daily; OD-once daily; QOD-every alternate day* (Adapted from Lacouture et al^16^)
